# Annotations

**Published:** 1890-05-17

**Authors:** 


### Annotations.

**A Parson on Doctors.**

The Rev. H. R. Haweis reads the doctors a plain-spoken little homily in the *Daily Graphic.* He wants to know why, since we have undoubtedly blundered about mesmerism, we have not had the straightforward manliness to say so? Mr. Haweis is both a philosopher and a man of science in his own peculiar way; and his philosophy enables him to answer his own question correctly. He says that doctors resist the admission of their error, not because they are doctors, but because they are men. Quite right, Mr. Haweis! You would probably be the very last to deny that ecclesiastics have occasionally been known to take up false positions and to stick to them in spite both of logic and of fact. Medical men, however, are in a kind of penitent mood just now. The sins of their professional ancestors lie heavy upon their consciences. The younger men, too, are gradually finding out that the teachers of ten, fifteen, or twenty years ago were by no means so infallible as they thought themselves. Nay, more, some of the better educated among the newly-fledged doctors and senior medical students are beginning to wonder whether medicine and physical science generally is really in every case as clearly demonstrable as the proposition that a square is a figure having all its sides equal and all its angles right-angles. In truth, it is now beginning to dawn upon doctors as a matter of practical business that there may conceivably be more things in heaven and earth than are dreamt of in their philosophy. Mr. Haweis rates us not only for declining to believe in Mesmer and his theories, but also for refusing to accept his facts. He practically charges us with meanly sneaking out of a false position instead of honestly and manfully describing a right-about-face. Hypnotism, he says in effect, is mesmerism under another name. Doctors are eager to lay hold of hypnotism, though not one in a thousand is willing to admit that he and his predecessors treated Mesmer more than badly. Some day or other a chapter will be written on unscientific scientists. It may be hoped that a doctor will write it, because he will be able to tell his professional brethren some plain truths which they ought to know. Here, for example, is a fact which makes one's professional ears tingle: In 1846 Dr. Elliotson, a famous practitioner with a large practice, was Harveian orator for the year. In the course of his oration he ventured to declare his belief in mesmerism. The doctors throughout the country at once and simultaneously denounced him, and that so thoroughly, that his practice was destroyed, and he soon after died, a heart-broken and practically ruined man. Now if those doctors who denounced Elliotson had known anything at all about the matter one would have excused their zeal. But the undeniable truth is that nine out of ten of them were as ignorant of mesmerism as they were of the Milky Way. We are obliged to admit that doctors are still human, and some of them are apt to speak with most authority on subjects they know least about. The history of mesmerism may properly furnish a warning to us all.

**May and Missionaries.**

Perhaps no one in the world needs to be so tenderly pitied, and so charitably considered, as the poor, unfortunate creature who is doomed to earn coats and trousers and bread and house rent by the art of making "fun." Even the missionary societies that are now meeting in Exeter Hall may well forgive, and drop a sympathetic tear upon the hand of the artist and the paragraph maker who secures a miserable five shillings by caricaturing and ridiculing their work. After all, we should remember who and what those same caricaturists and paragraphists are! They do not represent science, nor art, nor literature, nor society, nor business, nor, indeed, anything but a few poor hard-up journalists, who are driven to their wits' end for materials for fresh copy. The poor creatures do not know that the world is turned upside down since the days of John Leech. Darwin and Spencer have successively occupied the throne of philosophic science, and Stanley has brought the whole of Africa and its inhabitants close to our own shores. Development as an idea is in the air, and hundreds of ardent young men of the period are anxious to behold it in actual operation. It has come therefore to this, that if the missionary societies did not send out men to teach morality and religion to the backward peoples of the earth, scientific societies would be started which would send out missionaries of philosophy and science. But everything must come in its order. Religion is a much more fundamental and universal human endowment than the taste and aptitude for speculative truth. What an extraordinary thing it is that even philosophers who can follow so intelligently the lines of physical development, and can see so clearly the results of environment among the lower creatures, do not, nay, will not, recognise the plain and evident facts of moral and religious development as seen in every race of man and at every period of human history! Religion is at one and the same time the basis and the pioneer of civilisation, of mental and moral growth, and of all the highest intellectual developments of which man is capable. If the scientist and the philosopher understood the bearings of their own principles half as clearly and thoroughly as they ought to do, they would be the most ardent of all the supporters of the missionary societies. For our part we venture to offer to those societies our sincere gratitude, both from a scientific and a humanitarian standpoint. Every good, honest, and intelligent Briton put down in Africa, or other uncivilised country, is worth infinitely more than his weight in gold to the poor creatures to whom he carries literature, civilisation, and more of the divine light that lighteth every man that cometh into the world.

**"Wake Up, Mary."**

A few days ago the supporters of the Ragged School Union held their annual meeting at Exeter Hall, and Lord Kinnaird was in the chair. The Princess Mary of Cambridge had promised to be present at a certain stage of the proceedings to distribute the prizes. The hour came, but not the Princess. By way of filling up the time Lord Kinnaird had a previous song rendered over again; but still her Royal Highness did not come. After a long and expectant pause, however, she duly arrived, and took her place on the platform. The programme was thereupon proceeded with. But what was Lord Kinnaird's consternation to find that the very next item on the paper was a song, having the exquisitely appropriate title of "Wake up, Mary." The song was specially designed to impress on all and sundry the grievous sin of unpunctuality. Lord Kinnaird was, however, equal to the occasion, and, adroitly passing over the unlucky song, boldly called for the next, amid the amused laughter of his audience. We do not know whether this classical composition adjures "Mary" to "wake up" early in the morning; but we do know that early waking with early rising seems to be going as completely out of fashion as poke bonnets and umbrellas made of gingham. It is quite the common custom at the present time to "turn night into day and day into night." The custom has nothing to recommend it. It is always and everywhere and altogether bad. Perhaps if one were to say it shortens life it would be considered a very ineffective expostulation, because most people nowadays seem to be unable to look forward, even for a single year, much less for a whole lifetime. But late hours and late rising seriously injure the health as well as shorten the life, and they play dreadful havoc with good looks. We are going back to the time of Queen Anne, and even to that of Elizabeth, in the fashions of our houses. It would be much more to the point if we could go back in our habits of eating, sleeping, and taking exercise. The frivolous world will, no doubt, do as it pleases. What other conceivable thing, indeed, can it be expected to do? But those men and women of all classes and of every degree of culture who must do a day's work for each day will greatly advantage themselves by remembering that early rising and early retiring are conducive to sound sleep, and that of all the natural endowments which make for happiness and final success in life, sound sleep is, perhaps, the chief. "Wake up, Mary," should henceforth become a household word in every loyal British home.

